# Mumps Postexposure Prophylaxis with a Third Dose of Measles-Mumps-Rubella Vaccine, Orange County, New York, USA

**DOI:** 10.3201/eid1909.130299

**Published:** 2013-09

**Authors:** Amy Parker Fiebelkorn, Jacqueline Lawler, Aaron T. Curns, Christina Brandeburg, Gregory S. Wallace

**Affiliations:** Centers for Disease Control and Prevention, Atlanta, GA, USA (A. Parker Fiebelkorn, A.T. Curns, G.S. Wallace);; Orange County Health Department, Goshen, New York, USA (J. Lawler, C. Brandeburg)

**Keywords:** mumps, postexposure prophylaxis, third-dose MMR vaccine intervention, measles-mumps-rubella vaccine, viruses

## Abstract

Although the measles-mumps-rubella (MMR) vaccine is not recommended for mumps postexposure prophylaxis (PEP), data on its effectiveness are limited. During the 2009–2010 mumps outbreak in the northeastern United States, we assessed effectiveness of PEP with a third dose of MMR vaccine among contacts in Orthodox Jewish households who were given a third dose within 5 days of mumps onset in the household’s index patient. We compared mumps attack rates between persons who received a third MMR dose during the first incubation period after onset in the index patient and 2-dose vaccinated persons who had not. Twenty-eight (11.7%) of 239 eligible household members received a third MMR dose as PEP. Mumps attack rates were 0% among third-dose recipients versus 5.2% among 2-dose recipients without PEP (p = 0.57). Although a third MMR dose administered as PEP did not have a significant effect, it may offer some benefits in specific outbreak contexts.

Mumps is an acute, viral illness that classically is manifested as parotitis and can cause severe complications, including encephalitis (*1*), deafness (*2*,*3*), and orchitis (*4*). In 1977, the Advisory Committee on Immunization Practices (ACIP) recommended 1 mumps vaccine dose for routine childhood vaccination, and in 1989, the committee recommended that 2 doses of measles-mumps-rubella (MMR) vaccine be given to school-aged children and select high-risk groups for improved measles control (*5*). ACIP does not recommend administering MMR vaccine during mumps outbreaks as postexposure prophylaxis (i.e., vaccine administered during a brief window after exposure to prevent mumps infection) (*5*). Antibody response to the mumps component of MMR vaccine is generally believed to develop too late to provide effective prophylaxis after a person has been exposed to suspected mumps (*6*,*7*), but data are insufficient for to assessing a possible prophylactic effect.

During 2009–2010, a large mumps outbreak affected 3,502 persons in the Orthodox Jewish community in the northeastern United States. Students, from elementary school through college, had 2,370 (67.7%) cases; of these case-patients, 85% had received the recommended 2 doses of MMR vaccine (*8*). Yeshivas (i.e., private, traditional Jewish schools with extended school days) and households characterized by large families, typical in the Orthodox Jewish community, were the primary settings for mumps transmission (*8*). The objective of this study was to assess secondary mumps attack rates among Orthodox Jewish household contacts in Orange County, New York, who received PEP with a third dose of MMR vaccine within 5 days of mumps introduction into a household by a family member, and compare them with household contacts of persons who had received 2 previous MMR doses and did not receive PEP.

## Methods

### Study Population

The study population was a geographically and socially clustered community of ≈20,000 persons, primarily Orthodox Jews, in Orange County, New York. A common feature of this community was its high household contact rates because of large family size (average, 6 members) and shared bedrooms (*9*). Most members of this community followed ACIP vaccination recommendations; 2-dose MMR vaccine coverage among school-aged children in the community was 94.3%, which was higher than the national average (*8*,*10*,*11*).

### Case Definitions and PEP Definition

Mumps cases were classified according to the case definition for mumps of the Council of State and Territorial Epidemiologists in 2008 (*12*). Household members were considered secondary case-patients if mumps onset occurred 12–25 days (1 incubation period) after parotitis onset in the household’s index case-patient. Household members were considered to be co-primary case-patients if mumps onset occurred within 11 days after onset in the household index patient. PEP was defined as a dose of MMR vaccine given to a household contact within the first 5 days of another household member’s onset of provider-diagnosed mumps parotitis. Any dose administered earlier than this was not considered a PEP dose.

### Study Design and Eligibility Criteria

Suspected cases of mumps within the affected community were reported to the local health department, and parents were encouraged to contact one of the community’s 2 primary medical providers. The provider invited the parent to bring the case-patient and all other household members to the clinic and instructed the parent regarding routine measures required by the practice to prevent patients with respiratory illnesses from causing the other patients to be exposed to the virus. At the initial visit, the provider assessed the case-patient, and if a diagnosis of mumps was confirmed, the provider determined whether other household members had a history of mumps and their vaccination status. If the family visited the healthcare provider during the study period, February 24–April 24, 2010, then household members who met the eligibility criteria (i.e., had received 2 documented doses of MMR vaccine, had no contraindications for vaccination, had no history of mumps, and 5 days had not yet elapsed since onset of parotitis in the household index case-patient) were offered a third dose of MMR vaccine. Household members who were not up to date with their routine vaccinations were offered a first or second dose of MMR vaccine as PEP. Adult household members whose vaccination history was not documented were eligible to receive a dose.

Eligible family members who did not receive PEP either chose not to be vaccinated or lived in a household in which mumps had been diagnosed in a case-patient earlier in the outbreak, and it was too late for family members to receive PEP. Household members were not eligible for PEP and were excluded from the analysis if they had received a recent MMR vaccine dose within the past 60 days (i.e., either at their health care provider’s office or by participating in a recent school-based third-dose MMR intervention study [*10*]), if they had a history of mumps, if they were too young to be vaccinated (i.e., <1 year of age), or if they were a co-primary or index case-patient. Members of households who chose not to be vaccinated and members of households of mumps case-patients identified earlier in the outbreak who were not offered a third MMR dose were used as a comparison group.

Because the use of a third dose of MMR vaccine is not recommended by ACIP for PEP, a protocol was submitted and approved by the US Centers for Disease Control and Prevention and New York State Institutional Review Boards. Participants provided written consent or assent.

### Baseline and Follow-up Surveys

Baseline surveys captured demographic characteristics, MMR vaccination history, and mumps history of household members. Follow-up surveys were completed at least 60 days after the date of parotitis onset for the household mumps index case-patient; information gathered included any MMR doses received by family members since the baseline interview and whether mumps developed in any household members.

### Vaccination Status Verification

Vaccination status of study participants was assessed. Health care provider records were reviewed to verify this information. 

### Data Analysis

All data were analyzed with SAS 9.3 (SAS Institute Inc., Cary, NC, USA). For each household, we added the number of family members eligible for PEP with MMR vaccine and the number who received PEP. χ^2^ and Wilcoxon rank sum tests were used to compare 1) demographic characteristics and intervals since last MMR dose among index case-patients and 2) household members who received a third dose of MMR vaccine as PEP with persons who had 2 previous doses and did not receive PEP. Secondary mumps attack rates during the first incubation period after mumps onset in the index case-patient were calculated.

## Results

### Characteristics of the Index Case-Patients

Of the 49 index case-patients, 25 were male (51.0%) (Table 1). The median age was 9 years (range 1–39 years). Thirty-two (65.3%) had received 2 doses of MMR vaccine. Eleven (22.4%) index case-patients were unvaccinated or had unknown MMR vaccination status. Among the 38 (77.6%) who reported receiving >1 doses of MMR vaccine, the median interval since their last dose was 47 months (range 3–170 months).

**Table 1:** Characteristics of index case-patients with mumps, Orange County, New York, 2009–2010*

Characteristic	No. (%)
Age. y	
Median (range)	9 (1–39)
0–3	3 (6.1)
4–6	8 (16.3)
7–17	27 (55.1)
>18	11 (22.4)
Sex	
F	24 (49.0)
M	25 (51.0)
No. MMR doses	
0	11 (22.4)
1	5 (10.2)
2	32 (65.3)
3	1 (2.0)†
>1	38 (77.6)
Median interval (range) since last dose, mo	47 (3–170)

*MMR, measles-mumps-rubella vaccine; values are in no. (%) unless otherwise indicated. †Person received a third MMR does in January 2010 prior to parotitis onset in February 2010. This person did not receive the third dose as part of the study.

### PEP

In 49 households, there were 365 household members, of whom 239 (65.5%) were eligible to receive PEP and 126 (34.5%) were deemed ineligible and excluded from further analysis of mumps risk factors. Those excluded were the following: 59 household members who had received a recent dose of MMR vaccine within 60 days before the intervention, 49 who were the household index patients, 15 who were <1 year of age, 2 who became co-primary case-patients, and 1 woman with a history of having had mumps in 1979 (Figure).

**Figure:**
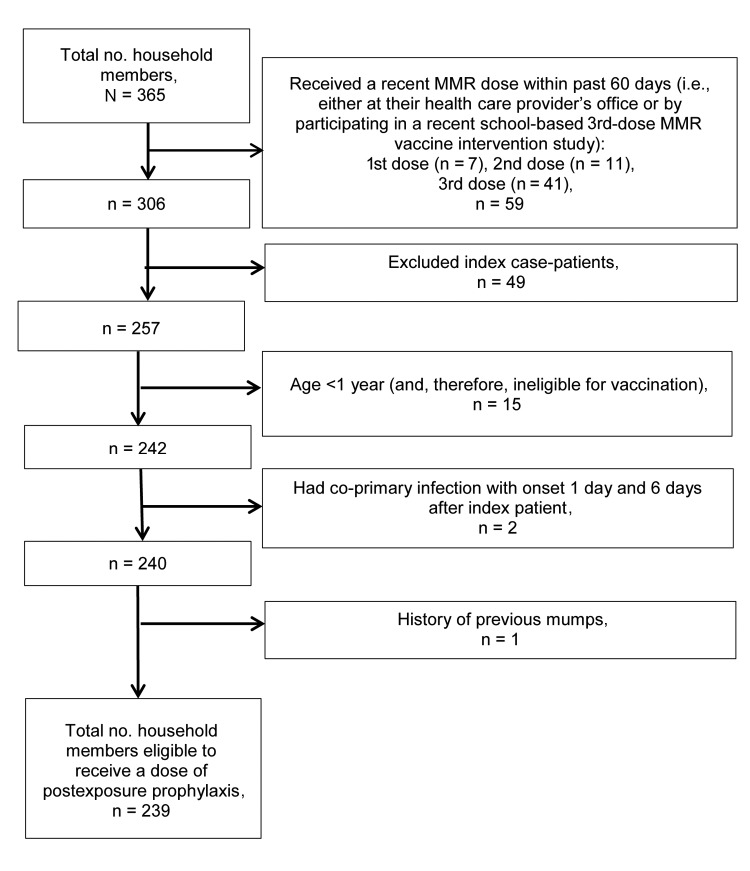
Exclusion criteria for household members eligible to receive a dose of measles-mumps-rubella (MMR) vaccine as postexposure prophylaxis, Orange County, New York, USA, 2009–2010.

Forty-four (18.4%) of the 239 eligible household members received a postexposure dose of MMR vaccine; 28 (11.7%) received a third MMR vaccine dose, 6 (2.5%) received a second MMR vaccine dose, 2 (0.8%) received a first MMR vaccine dose, and 8 (3.3%) adults with unknown vaccination status received a dose. The age groups of household members who received a third dose of MMR vaccine as postexposure prophylaxis included 10 (27.8%) of 36 children aged 4–6 years, 17 (24.3%) of 70 children 7–17 years of age, and 1 (1.1%) of 88 adults >18 years. Of the 16 other household members who received PEP with MMR vaccine, 2 children 1 year of age received a first dose, 6 children 1–17 years of age received a second dose, and 8 adults with unknown MMR vaccination status received a dose (Table 2).

**Table 2:** Classification by age group of eligible family members who did or did not receive a dose of MMR vaccine as postexposure prophylaxis, Orange County, New York, USA, 2009–2010*

Age, y	No. received 3rd MMR dose	No. received 2nd MMR dose	No. received 1st MMR dose	No. that received any dose (dose unknown)†	No. that did not receive any dose	Total no. eligible
1–3	0	3	2	0	39	44
4–6	10	2	0	0	24	36
7–17	17	1	0	0	52	70
>18	1	0	0	8	79	88
Unknown	0	0	0	0	1	1
Total (%)	28 (12)	6 (3)	2 (1)	8 (3)	195 (82)	239

*MMR, measles-mumps-rubella vaccine. †MMR vaccine doses were administered as postexposure prophylaxis.

Postexposure vaccinations were not administered to 195 (81.6%) eligible household members. Of eligible persons who did not receive postexposure vaccine, 77 had previously received 2 doses (of whom 21 were 4–6 years of age, 50 were 7–17 years, and 6 were >18 years), 40 had previously received 1 dose (of whom 33 were 1–3 years, 3 were 4–6 years, 2 were 7–17 years, and 2 were >18 years), and 78 had unknown vaccination status (of whom 6 were 1–3 years of age, 71 were >18 years, and 1 was <18 years, but the exact age was not available).

### Secondary Case-Patients

Of the 9 household secondary cases that occurred during the first incubation period after the index patient’s mumps onset, 3 (33.3%) were in male patients. Only 1 (11.1%) case-patient received the MMR vaccine as PEP. He was a 27-year-old father with unknown vaccination status. Eight (88.9%) persons who did not receive PEP became infected with mumps during the first incubation period after their exposure (2 were from the same household).

The median age of the 8 secondary case-patients who did not receive PEP was 18.5 years (range 6─39 years). The ages and vaccination status of these 8 included the following: 1 child 6 years of age who had a history of 2 doses of MMR vaccine, 3 children 7–17 years of age who had a history of 2 doses of MMR vaccine, and 4 adults with unknown vaccination status. All household members >18 years of age who were infected were parents of index case-patients. The interval between the last MMR vaccine dose and reported mumps onset was 18 days for the father who received PEP and from 2 to 6 years for the 4 case-patients with known vaccination status who did not receive PEP.

Mumps also developed in 2 persons within the first 11 days of the onset of the index case; these patients were considered co-primary. No secondary cases developed during the first incubation period after the index patient’s mumps onset among remaining family members at risk for mumps in the 2 households with co-primary case-patients (5 members were at risk in each household).

The interval between receipt of the last dose of MMR vaccine and mumps onset among the index case-patients did not differ between households with a secondary case-patient and those without (median interval 3 years; both groups, p = 1.0). Additionally, the ages of the index case-patients did not significantly differ between households with a secondary case-patient and those without (median ages 7.5 years and 9 years, respectively; p = 0.21).

### Persons Who Received Third Dose of MMR Vaccine versus Those Who Received 2 Doses

None of the 28 family members who received a third dose of MMR vaccine as PEP became infected with mumps virus in contrast with 4 (5.2%) of the 77 who had previously received 2 doses but did not receive PEP (Table 3). The difference in secondary attack rates between the 2 groups was not statistically significant (p = 0.57). Two of the 2-dose case-patients were male; the sex-specific attack rates were 6.9% for male and 4.3% for female patients (p = 0.62). The median age of those receiving a third dose was 8 years (range 5–20 years) and also 8 years (range 4–20 years) among those eligible who did not. The median number of years since the last MMR dose (before the PEP dose) was 10 years (range 2–39 years) among those who received a third dose, compared with 11 years (range 0–39 years) among those eligible who did not (p = 0.47).

**Table 3:** Demographic characteristics, median number of months since second MMR vaccine dose, and number of mumps case-patients among household members, Orange County, New York, USA, 2009–2010*

Characteristic	Received third MMR dose as PEP, n = 28	Had 2 previous MMR vaccine doses, received no PEP, n = 77	p value
Sex			
M	16 (57.1)	29 (37.7)	0.19
F	12 (42.9)	47 (61.0)	
Unknown	0	1 (1.3)	
Age, y			
4–6	10 (35.7)	21 (27.3)	0.58
7–17	17 (60.7)	50 (64.9)	
>18	1 (3.6)	6 (7.8)	
Median no. months since 2nd MMR dose, IQR	120 (62–177)	139 (62–210)	0.47
Minimum–maximum no. months	32–468	10–468	
Mumps onset, attack rate†	0	4 (5.2)	0.57

*MMR, measles-mumps-rubella vaccine; PEP, postexposure prophylaxis; IQR, interquartile range; values are no. (%) unless otherwise indicated. †Onset of mumps occurred 12–25 days after onset of mumps in index case-patient.

## Discussion

Although the attack rate among persons who received a third dose of MMR vaccine as PEP was 0%, compared with a 5.2% attack rate for those with 2 doses who did not receive PEP, the difference was not statistically significant. Nonetheless, MMR vaccine administered because PEP might offer some benefits. If the exposure did not result in infection, the vaccine should boost antibody titers high enough to induce protection against subsequent infection (*13*,*14*). Such boosting of antibody titers would be useful during an outbreak in which the virus continues to circulate and future exposures are likely. If infection does occur, the postexposure vaccine dose may lead to milder clinical manifestations, lower complication rates, and shorter duration of virus shedding (*15*).

Although a third dose of MMR vaccine has been previously administered for outbreak control (*10*,*16*), to our knowledge, a third dose of MMR vaccine has never previously been administered in a study to assess its efficacy as PEP. In 1986, a first dose of MMR vaccine was given as PEP in a Tennessee public high school to 53 of 178 students with no presumptive evidence of immunity. During the Tennessee outbreak, in 15 (28.3%) of 53 students who received a first dose of MMR vaccine as PEP, mumps developed between 1 and 21 days (1 incubation period) after they visited the clinic compared with mumps developing in 51 (40.8%) of 125 nonvaccinated students who did not receive PEP (*6*)..

In addition to the outbreak in the northeastern United States, other large mumps outbreaks have occurred among highly vaccinated US populations in recent years. In 2006, a total of 6,584 reported cases occurred, primarily in college students in the midwestern United States. Standard control measures (e.g., isolation and vaccine catch-up campaigns) were implemented for outbreak control (*17*) with modest effectiveness. The outbreak did not subside until summer break when the students left their college campuses. During 2009-2010, a total of 505 mumps cases were reported in the US Territory of Guam, primarily among school-aged children 9–14 years of age, 96% of whom had received 2 doses of MMR vaccine. In addition to application of standard control measures, a third dose of MMR vaccine was administered to the most affected age group for outbreak control, not as PEP. The effectiveness of the intervention was inconclusive (*16*). Outbreaks have also been reported in other industrialized countries among populations in which the proportion who received 2 doses of vaccine was high (*18*–*21*).

Two MMR vaccine doses provide 66%–95% effectiveness against mumps (*22*,*23*), and the 2-dose policy has reduced mumps incidence by >99% compared with incidence during the prevaccine era (*24*). Nonetheless, mumps outbreaks in well-vaccinated populations continue to occur, posing challenges for outbreak control. Current public health measures for preventing the spread of mumps during outbreaks, including isolation, quarantine, contact tracing, and increasing vaccine coverage have had limited effect (*17*,*25*). When schools follow public health guidance and send infected students home for 5 days, the intervention may be too late. Mumps can spread from symptomatic persons before parotitis onset. Mumps can also spread from persons who have asymptomatic infections, which can be as high as 15%–27% of infected persons (*4*,*26*). In addition, isolating patients and quarantining contacts may be ineffective when infected persons live in large households with many other susceptible persons. Finally, raising vaccine coverage is also difficult in contexts where 2-dose vaccine coverage is already high, because current policy does not recommend a routine third MMR vaccine dose (*5*).

In the 2 households with co-primary cases in this study, no additional cases occurred during the first incubation period. This finding suggests that those households were not more infectious than households with only 1 index patient.

This study was subject to limitations, however. Household members may have been exposed to mumps by a contact outside the home. Although our method might have been more robust if we could have randomly selected household contacts to receive PEP, because of ethical considerations, it was necessary to offer PEP to all eligible household contacts. Some household members had received the third dose during a school intervention a couple of months before this study. In addition, some members received either a first, second, or third dose during the outbreak but not as part of the study. Although these persons were excluded from the analysis because their doses were not administered as PEP, these doses outside the study may have limited the effect of the study doses because additional family members were protected. This could have lowered mumps attack rates in the households by reducing the number of susceptible persons. When the risk for mumps among persons potentially susceptible was assessed, the limited sample size and low attack rates resulted in large confidence intervals. Finally, the power of the study to detect a significant difference was extremely low because of the small number of study households, the relatively late implementation of the study during the outbreak, and the low number of mumps cases that occurred in the study population.

Although 2 MMR doses are sufficient for preventing mumps in most settings, administering a third MMR dose may be worthwhile in specific outbreak contexts, even if it does not offer protection as PEP. Our findings support the need for additional evaluations in which third doses of MMR vaccine are used as PEP in outbreaks among populations with 2-dose vaccination coverage. Future studies on administering any dose of MMR vaccine for mumps PEP during mumps outbreaks are also warranted.
